# [Retracted] miR‑30a‑5p inhibits the proliferation, migration and invasion of melanoma cells by targeting SOX4

**DOI:** 10.3892/mmr.2023.13071

**Published:** 2023-08-14

**Authors:** Erbiao Liu, Xiyan Sun, Jinping Li, Chao Zhang

Mol Med Rep 18: 2492–2498, 2018; DOI: 10.3892/mmr.2018.9166

Following the publication of this paper, it was drawn to the Editor's attention by a concerned reader that the tumour images shown in Fig. 3B were strikingly similar to data appearing in different form in another article written by different authors at different research institutes. Owing to the fact that the contentious data in the above article were already under consideration for publication prior to its submission to *Molecular Medicine Reports*, the Editor has decided that this paper should be retracted from the Journal. The authors were asked for an explanation to account for these concerns, but the Editorial Office did not receive a reply. The Editor apologizes to the readership for any inconvenience caused.

